# Marqueurs chromosomiques: à propos d'un cas

**DOI:** 10.11604/pamj.2013.15.104.1993

**Published:** 2013-07-18

**Authors:** Imane Samri, Laila Bouguenouch, Hasna Hamdaoui, Ihsan El Otmani, Nissrine El Omairi, Sana Chaouki, Moustapha Hida, Karim Ouldim

**Affiliations:** 1 Unité de Génétique Médicale et d'Oncogénétique. Laboratoire Central d'Analyses Médicales, CHU Hassan II, Fès, MAROC; 2 Service de Pédiatrie CHU Hassan II, Fès, Maroc

**Keywords:** Marqueurs chromosomiques, anomalie chromosomique, analyse cytogénétique, dysmorphie

## Abstract

Les marqueurs chromosomiques peuvent être définis comme des petits chromosomes de structure anormale présents en addition aux 46 chromosomes humains connus. C'est un groupe hétérogène d'anomalies de structure chromosomique pouvant être avec ou sans conséquence phénotypique. Plusieurs tentatives sont réalisées afin de retrouver une corrélation génotype-phénotype lors de la présence d'un marqueur chromosomique. L'identification du marqueur, son origine et sa structure suit une stratégie bien codifiée actuellement allant d'abord de l'orientation clinique suivie des techniques de cytogénétique conventionnelle (caryotype métaphasique standard, bandes C, NOR) et de cytogénétique moléculaire (M-FISH, CGH, CGH array) puis une détection par des techniques plus ciblées (painting, sondes locus spécifique). Cet ensemble permet une meilleure analyse et correspondance clinico-génétique. Nous rapportons le cas d'un nourrisson présentant une dysmorphie faciale avec un retard psychomoteur dont l'analyse cytogénétique a révélé la présence d'un marqueur chromosomique avec un caryotype métaphasique 47,XX,+mar. A travers cette observation, nous mettons en valeur le rôle de la cytogénétique conventionnelle et moléculaire dans le diagnostic des syndromes dysmorphiques permettant une meilleure prise en charge du patient et un conseil génétique adéquat pour sa famille

## Introduction

Un marqueur chromosomique surnuméraire est un chromosome additionnel de structure anormale dont l'origine et la composition ne peut être détectée par les techniques de cytogénétique conventionnelle. Ils peuvent dériver de tous les chromosomes humains, autosomes et gonosomes, avec une fréquence plus marquée à partir des chromosomes acrocentriques. La fréquence de cette anomalie de structure est estimée entre 0.028% et 0.15% avec un pourcentage de 30% de l'origine parentale. La présentation clinique des MCS est d'une grande variabilité: certains syndromes sont décrits en relation avec certains MCS: le syndrome de Pallister-Killian, le cat-eye syndrome, la tétrasomie 18p. Cependant d'autres MCS sont sans conséquence phénotypique d'où l'intérêt d’étudier cette anomalie afin d’établir une bonne corrélation génotype phénotype. L'analyse des MCS nécessitent le recours aux techniques de cytogénétique conventionnelle en premier lieu afin d'orienter la stratégie moléculaire d'identification alors que l’étude spécifique de l'origine et la structure des MCS utilisent des techniques de cytogénétique moléculaires plus adaptées telles que la multiplex-FISH(M-FISH) et le caryotype spectral (SKY) et plus récemment la CGH et la CGH array afin de détecter la présence ou non d'euchromatine sur les MCS. A travers ce cas, nous mettons à jour les actualités scientifiques de cette anomalie chromosomique ainsi que les différentes techniques et stratégies de son identification permettant une prise en charge adaptée au cas par cas et un suivi et conseil génétique adéquat.

## Patient et observation

**Figure 1:**
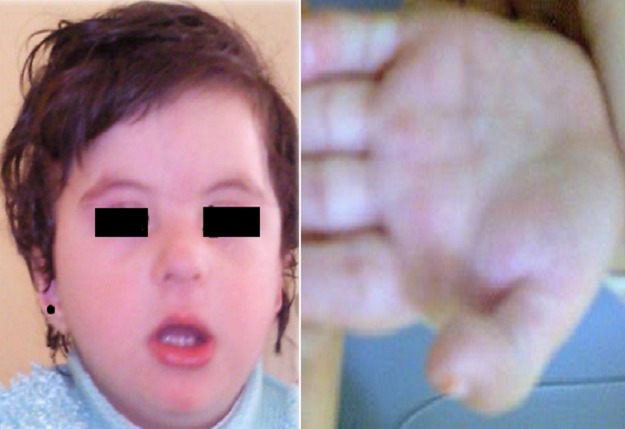
Phénotype de la patiente H.A

Notre patient est un nourrisson âgée de 2 ans et demi, de sexe féminin adressée en consultation de génétique médicale pour syndrome dysmorphique et retard mental. L'enfant est issu d'un mariage non consanguin, père âgé de 54 ans et mère âgée de 46 ans, 3ème d'une fraterie de 3, d'une grossesse non suivie estimée à terme sans incidents. Accouchement par voie basse médicalisé avec une hospitalisation pendant 5 jours pour souffrance néonatale. L'enfant est opérée à l’âge de 1 an pour luxation congénitale de la hanche. Un retard des acquisitions psychomotrices est présent: position assise à 9 mois, position debout à 2 ans, marche non encore acquise avec retard du langage. Poids et taille normaux. Le syndrome dysmorphique est fait de: hypertélorisme, oreilles bas implantées, lèvre supérieure fine, pli palmaire transverse unique. Les doigts de la main gauche ont une taille presque égale et sont tous déformés en col de cygne. Au niveau des membres inférieurs un genu varum droit est noté (Figure 1). Le bilan malformatif est sans particularité notamment la tomodensitométrie cérébrale, l’échographie cardiaque et l’échographie abdominale. Le bilan biologique a objectivé une TSHus légèrement supérieure à la normale. Les parents sont phénotypiquement normaux avec absence de cas similaires dans la famille.

Le caryotype métaphasique est réalisé à partir de cultures de lymphocytes à 37°C pendant 72h. Les cellules sont bloquées en métaphase par la colchicine. Après un choc hypotonique au KCI, les mitoses sont fixées par un mélange méthanol/acide acétique. Les préparations chromosomiques ainsi obtenues sont dénaturées par la chaleur (bandes RHG) et colorées au Giemsa. Onze mitoses sont prises en photo, puis classées en fonction de la taille des chromosomes, de leur indice centromérique et de la succession des bandes, de façon semi-automatique grâce à un logiciel spécialisé couplé à une caméra.

**Figure 2:**
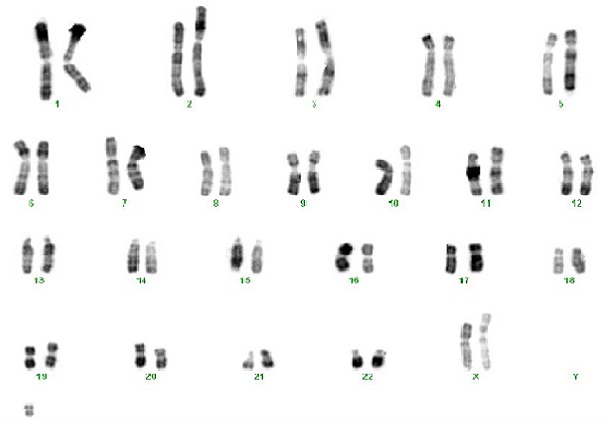
Caryotype métaphasique en bandes R: 47,XX,+mar

Le caryotype métaphasique en bandes R de notre patient a mis en évidence la présence d'un marqueur chromosomique (Figure 2) 47,XX,+mar sur les onze mitoses analysées. L'analyse du caryotype des parents (indispensable en cas d'anomalies de structure) n'a pas été réalisé vue le refus des parents de poursuite des autres investigations diagnostiques.

## Discussion

Les marqueurs chromosomiques surnuméraires (MCS) sont définis par Liehr et al., comme des chromosomes de structure anormale et qui ne peuvent pas être identifiés par les techniques de cytogénétique conventionnelle seules, ils ont une taille égale ou inférieure au chromosome 20 de la même préparation métaphasique. En revanche, un MCS de taille supérieure au chromosome 20 est retrouvé dans certaines bases de données. Ils peuvent être présents dans un caryotype normal à 46 chromosomes, un caryotype avec anomalie de nombre (ex : trisomie 21) ou dans un caryotype avec anomalie de structure mais équilibrée (ex : translocation robertsonienne ou chromosome en anneau).

Les MCS peuvent dériver de tous les chromosomes, autosomes et gonosomes, avec une fréquence plus importante des dérivés des chromosomes acrocentriques et plus particulièrement les chromosomes 15 et 22.

**Figure 3:**
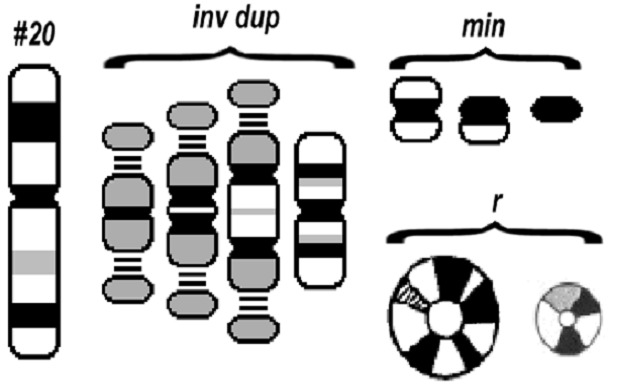
Différentes structures des marqueurs chromosomiques

La structure des marqueurs chromosomiques surnuméraires est également extrêmement variable: dérivés (der), inversion duplication (inv dup), anneau (r), isochromosomes (i), chromosomes minutes (min) (Figure 3). Ainsi, la description des MCS comme «marqueurs», fait sens et devrait être maintenue, même après leur identification par cytogénétique moléculaire.

Les marqueurs chromosomiques surnuméraires, désignés« +mar » dans la nomenclature internationale [1], sont des anomalies dont la fréquence est estimée entre 0.024 et 0,219 % en moyenne 0.044%. 70%des MCS sont de novo et dans cette catégorie 74% sont sans conséquence phénotypique(2).Et si le marqueur est hérité, le risque de manifestation clinique est plus faible.

Les personnes portant un MCS, ont une large gamme de variabilité clinique qui peut être due à la différence de l'origine des MCS, leur taille, la présence et / ou l′absence de matériel euchromatique, le degré de mosaïcisme et / ou une disomie uniparentale (UPD) [3]. Les MCS sont retrouvés dans 0.288% en cas de retard mental, 0.125% en cas d'hypofertilité (hommes 0.165% versus femmes 0.022%) [3]. Mais certains MCS sont sans conséquence phénotypique [16]. Ils impliquent essentiellement les régions péricentromériques, les régions satellites des bras courts des chromosomes acrocentriques et généralement ne contiennent pas d'euchromatine.

La fréquence des MCS en prénatal est estimée à 0.075% pour des cas étudiés en raison de signes d'appel échographiques, anomalies biologiques (triple test) ou un âge maternel avancé. En effet, une incidence plus élevé en cas d’âge maternel avancé a été rapportée.

Plusieurs tentatives ont été faites pour corréler des MCS spécifiques à un tableau clinique. Cela a abouti à la description de quelques syndromes particuliers tels le syndrome de Pallister-Killian (l'isochromosome du bras court du chromosome12 : i(12)(p10)), le syndrome de l'isochromosome des bras courts du chromosome 18 (i(18)(p10)), le cat-eye syndrome (l'inversion duplication du bras long du chromosome 22 : inv dup(22)(q11)), le syndrome associé au dérivé du chromosome 22 (der(22)t(11;22)(q23;q11.2)). Cependant, la plupart des marqueurs n'ont pas été entièrement caractérisés. Cette entité d'anomalie chromosomique peut être homogène ou en mosaïque ce qui augmente le taux de variabilité clinique rendant la corrélation génotype-phénotype plus délicate. L'avènement de nouvelles techniques de cytogénétique moléculaire telles que la FISH et ses différents dérivés ainsi que la CGH ont permis de mieux caractériser l'origine et la structure de ces chromosomes additionnels.

Ces techniques d'analyse des MCS se présentent sous deux groupes: Le premier groupe permettant une approche globale des MCS comprenant la FISH multicouleurs (dite aussi la FISH 24-couleurs) comme technique de base, outil indispensable pour identifier l'euchromatine d'un chromosome marqueur surnuméraire. Cette technique permet grâce à l'hybridation de 24 sondes marquées par 5fluorochromes l'identification du chromosome à l'origine du MCS vue que chaque chromosome possède une combinaison de couleurs bien précise. Cependant, la multi FISH ne permet pas de déterminer la région impliquée. Ainsi d'autres dérivés de la FISH multicouleurs ont vue le jour afin de bien caractériser la région concernée. Parmi eux on cite : l'arm-FISH permettant d'identifier chaque bras des 24 chromosomes humains, en excluant les bras courts des chromosomes acrocentriques et du chromosome Y. la cenMFISH [5] spécifique des centromères est également une technique de FISH multicouleurs qui grâce à l'hybridation simultanée de tous les centromères détermine l'origine du MCS sans pour autant préciser la présence ou l'absence d'euchromatine. Starke et al.[4] ont développé la FISH multicouleurs subcentromérique (subcenMFISH), en utilisant un ensemble de sondes bacterial artificial chromosomes (BACs) et Yeast artificial chromosomes (YACs), localisées dans la région péricentromérique de chaque chromosome. La subcenMFISH est concurrente des méthodes précédentes pour la caractérisation des MCS. En 2001, l'accroMFISH a vu le jour permettant la détection simultanée d'hétérochromatine et d'euchromatine des chromosomes accrocentriques.

En 1992, la technique de la CGH puis sa dérivée la ȜCGHarray µ array comparative genomic HybridizationȜ, techniques basées sur la comparaison entre l'ADN d'un patient avec celui d'un témoin hybridés tous les deux sur des puces à ADN. Ainsi, par cet outil, il est possible de déterminer l'origine et la composition d'un MCS. A noter qu’à ce jour La CGH-array reste la technique la plus sensible pour l'identification des chromosomes additionnels.

D'autre part, le second groupe comporte des techniques d'analyse plus ciblées telle le banding multicouleur (m-Band ou MCB ou m-banding),hybridation de sondes fluorescentes dont le résultat est une succession de bandes fluorescentes de couleurs différentes sur une paire chromosomique donnée caractérisant ainsi les réarrangements intrachromosomiques mais cette technique nécessite la reconnaissance au préalable du chromosome d'origine du MCS. Aussi les sondes centromériques spécifiques (CEP) et les sondes locus spécifiques sont utilisées en deuxième intention pour confirmer l'origine chromosomique et/ou pour préciser la structure de l'anomalie.

**Figure 4:**
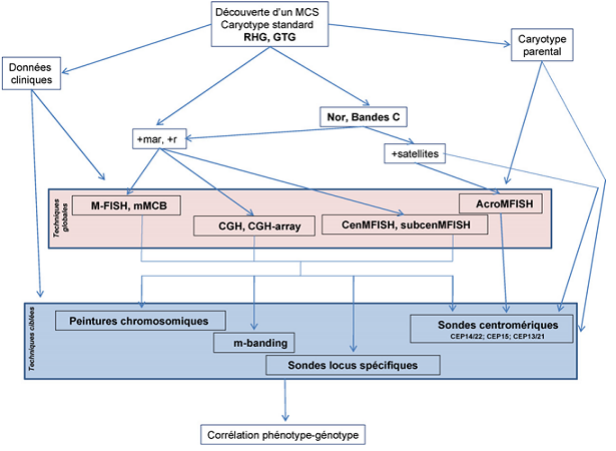
Stratégie d ‘identification d'un marqueur chromosomique surnuméraire

Devant la complexité de l'analyse du MCS afin de déterminer sa nature, son origine, sa structure et la présence ou non d'euchromatine d'une part et les multitudes des techniques présentées permettant son étude d'autre part, une stratégie doit être envisagée pour une meilleure approche et compréhension de cette anomalie chromosomique. La présentation clinique et les techniques de cytogénétique conventionnelle peuvent orienter la stratégie moléculaire d'identification. Pour l’étude moléculaire, il est préférable de commencer par une analyse globale, et la FISH 24-couleurs, la CGH et la CGH-array semblent les plus appropriées pour identifier la présence d'euchromatine d'un MCS. Quand le marqueur est de très petite taille ou présent en faible mosaïque, des techniques plus ciblées peuvent être utilisées (Figure 4)

L’étude cytogénétique d'un MCS doit être complétée par la recherche de disomie uniparentale si l'origine chromosomique concerne les chromosomes 6, 7, 11, 14, 15 et 20. Par exemple, pour les MCS du chromosome 15, la présence de la région du Prader- willi/ Angelman (identifiée avec la sonde locus spécifique) semble être corrélée à la sévérité du phénotype.

## Conclusion

Les marqueurs chromosomiques représentent une entité d'anomalies chromosomique d'une grande hétérogénéité et variabilité nécessitant une approche clinique, biologique et génétique plus approfondie avec un recueil de cas plus important afin d'identifier la nature et la structure des MCS, leur composition en euchromatine et son retentissement clinique et pouvoir élucider la corrélation génotype-phénotype encore mal connue permettant ainsi une prise en charge adéquate du patient et un conseil génétique personnalisé à sa famille.

## References

[1] Liehr T Homepage on small supernumerary marker chromosomes (SMC). http://www.med.unijena.de/fish/sSMC/00START.htm.

[2] Liehr T, Weise A (2007). Frequency of small supernumerary marker chromosomes in prenatal, newborn, developmentally retarded and infertility diagnostics. Int J Mol Med..

[3] Liehr T, Mrasek K, Weise A, Dufke A, Rodriguez L (2006). Small supernumerary marker chromosomes - progress towards a genotype-phenotype correlation. Cytogenet Genome Res..

[4] Starke H, Nietzel A, Weise A, Heller A, Mrasek K, Belitz B (2003). Small supernumerary marker chromosomes (SMCs): genotype-phenotype correlation and classification. Hum Genet..

[5] Nietzel A, Rocchi M, Starke H, Heller A, Fiedler W, Wlodarska I (2001). A new multicolor-FISH approach for the characterization of marker chromosomes: centromere-specific multicolor-FISH (cenM-FISH). Hum Genet..

[6] Liehr T, Ewers E, Hamid AB, Kosyakova N, Voigt M, Weise A, Manvelyan M (2011). Small supernumerary marker chromosomes and uniparental disomy have a story to tell. J Histochem Cytochem..

[7] Villa N, Bentivegna A, Ertel A, Redaelli S, Colombo C, Nacinovich R, Broggi F, Lissoni S, Bungaro S, Addya S, Fortina P, Dalprà L (2011). A de novo supernumerary genomic discontinuous ring chromosome 21 in a child with mild intellectual disability. Am J Med Genet A..

[8] Eckmann-Scholz C, Tönnies H, Liehr T, Gesk S, Jonat W, Caliebe A (2012). Normal prenatal ultrasound findings in a case with de novo mosaic small supernumerary marker chromosome 18 - how to counsel. J Matern Fetal Neonatal Med..

[9] Manolakos E, Kefalas K, Neroutsou R, Lagou M, Kosyakova N (2010). Characterization of 23 small supernumerary marker chromosomes detected at pre-natal diagnosis: The value of fluorescence in situ hybridization. Mol Med Rep..

[10] Sheth F, Andrieux J, Ewers E, Kosyakova N, Weise A, Sheth H, Romana SP, LeLorc'h M, Delobel B, Theisen O, Liehr T, Nampoothiri S, Sheth J (2011). Characterization of sSMC by FISH and molecular techniques. Eur J Med Genet..

[11] Mikelsaar R, Lissitsina J, Bartsch O (2011). Small supernumerary marker chromosome (sSMC) derived from chromosome 22 in an infertile man with hypogonadotropic hypogonadism. J Appl Genet.

[12] Chen CP, Chen M, Su YN, Tsai FJ, Chern SR, Wu PC, Chen WL, Chen LF, Pan CW, Wang W (2011). Prenatal diagnosis and molecular cytogenetic characterization of mosaicism for a small supernumerary marker chromosome derived from ring chromosome 4. Taiwan J Obstet Gynecol..

[13] Liehr T, Karamysheva T, Merkas M, Brecevic L, Hamid AB (2010). Somatic mosaicism in cases with small supernumerary marker chromosomes. Curr Genomics..

[14] Vorsanova SG, Yurov YB, Iourov IY (2010). Human interphase chromosomes: a review of available molecular cytogenetic technologies. Mol Cytogenet..

[15] Melo JB, Backx L, Vermeesch JR, Santos HG (2011). Chromosome 5 derived small supernumerary marker: towards a genotype/phenotype correlation of proximal chromosome 5 imbalances. J Appl Genet..

[16] Balkan M, Isi H, Gedik A, Erdemo'lu M, Budak T (2010). A small supernumerary marker chromosome, derived from chromosome 22, possibly associated with repeated spontaneous abortions. Genet Mol Res..

[17] Nelle H, Schreyer I, Ewers E, Mrasek K, Kosyakova N (2010). Presence of harmless small supernumerary marker chromosomes hampers molecular genetic diagnosis: a case report. Mol Med Rep..

